# Defining standards and core outcomes for clinical trials in prehabilitation for colorectal surgery (DiSCO): modified Delphi methodology to achieve patient and healthcare professional consensus

**DOI:** 10.1093/bjs/znae056

**Published:** 2024-06-18

**Authors:** Rebecca Fish, Sue Blackwell, Stephen R Knight, Sarah Daniels, Malcolm A West, Iona Pearson, Susan J Moug, DiSCO Study Group, Deborah S Keller, Nanette Mutrie, Paul Kelly, Nicola S Fearnhead, Carly Bisset, Jene Ng, May Thu, Mike Kelly, Adam Boutall, Aileen Schofield, Alastair McKay, Alec Mcdonald, Alex Labey, Alexander Heriot, Alexandros Charalabopoulos, Alice Finch, Alison Culkin, Alison Payne, Alistair Owen, Amanda Reid, Amy Kerr, Andrei Tanase, Andrew Dennis, Andrew Miller, Andrew Renehen, Andrew Renwick, Andrew Rogerson, Andriana Petrova, Angela Baker, Angeline Adams, Angeline Price, Angie Balfour, Anisa Kushairi, Ann-Maree Murphy, Anne Marks, Annelies Mittendorff, Annie McCaffery, Arman Erkan, Arnold Goede, Ash Bhalla, Asif Mehraj, Ayse Gizem Unal, Baljit Singh, Ben Griffiths, Beryl Goddard, Bill Campbell, Brian Devlin, Brian Power, Cain Clark, Caroline Dodds, Carolyn Atkin, Catherine Radley, Catriona Brown, Charles Hendrickse, Charlotte Atkinson, Charlotte Foley, Chelsia Gills, Chloe Beard, Chloe M Grimmett, Chloe Nottingham, Christopher J Smart, Claire Cruikshanks, Claire Finlayson, Claire Hall, Claire Knowles, Claire McCann, Claire Taylor, Clare E Collins, Clare Shaw, Colette Backwell, Craig Gilmour, Craig Lynch, Danella Ruddy, Daniel Santa Mina, Dave Pawson, David Easton, David Lubowski, David Oglesby, David Proud, David Shipway, David Watt, David Yates, Dean Harris, Debbie Mulhern, Deborah Howland, Deborah Keller, Deepa Muthukrishnan, Denny Levett, Dermot Burke, Duncan Miller, Effie Jamieson, Efstratia Baili, Eleanor Gray, Eleanor Hitchman, Eleftheria Douka, Emma Greenwood, Emma McMillan, Emma Nicholls, Evgeniy Drozdov, Fergal Fleming, Fiona Windsor, Fionna Martin, Francesco Maria Carrano, Franco Carli, Fraser Smith, Gary Howell, Gary Nicholson, Geert Koffeman, Gemma Faulkner, Gemma Ford, Georgina Giebner, Gianluca Pellino, Glen Guerra, Gregory Thomas, Heather Gilmore, Heather MacKinnon, Honor Blackwood, Hugh Paterson, Hwei Jene Ng, Hytham K S Hamid, Iain Jourdan, Ian Bissett, Ian Daniels, Isla Veal, Jackie Timperley, James Dilley, James Glasbey, James Holding, James Toh, Jamie Alcock, Jan Clarke, Jane Booker, Javier Ripollés-Melchor, Jeanette Osborne, Jeanette Preston, Jennie Burch, Jennifer Edwards, Jennifer Mackney, Jennifer Stewart, Jennifer Henderson, Jenny Pipe, Jenny Woodward, Jeremy Williamson, Jessica Bower, Jessica Mijnssen, Jo Lloyd, Joanna Flint, Joanne Logan, John Jameson, John Woodfield, Jon Lund, Jonathan Heath, John Moore, Jugdeep Dhesi, Julie Berson, Julie Wilkinson, June Davis, Justin Davies, Karen Kerr, Karen O’Hare, Karen Robb, Karen Telford, Karina Va Zquez-Narvaez, Karol Pal, Kathy Borthwick, Kausik Ray, Kellie Owen, Kenny Nattrass, Kerry S Courneya, Khalid Osman, Kimberley Adams, Kirsten Cassidy, Kirsty Rowlinson Groves, Kirsty Wade, Krishanthi Sathanandan, Krishna Kholia, Laura Hancock, Laura McGarrity, Lauren Coyle, Leah Cox, Lena W S Ngu, Leon Fu, Liam Humphreys, Linda Tutty, Liz Murphy, Lorraine Hughes, Louise Hunt, Louise Maxwell, Louise Perryman, Loukas Nadiotis, Luke Wheldon, Manisha Shah, Margaret Clark, Maria Burton, Marie Sheahan, Mark Bagnall, Mark Graham, Martin Rutegard, Mary Schactler, Marylise Boutros, Matthew J Lee, Mhairi Burke, Mhairi Simpson, Michael Davies, Michael P Kelly, Michael Lim, Michael Suen, Michele Carvello, Michelle Willcocks, Mike Grocott, Mohamed Shams, Monica Millan, Mukul Dube, Nadine Harran, Natalie Smith, Andrew Renehan, Omer Aziz, Nauman Ahmed, Neil Agnew, Neil Bibby, Neil Smart, Nicola Dames, Nicola Hill, Nicola Maguire, Nicola Peat, Nicole Saur, Nigel Horwood, Nigel Richardson, Nurulamin Noor, Peter Ishak, Peter Loder, Peter Murchie, Philip Walton, Pia Bernardi, Rachael Clifford, Rachel Hargest, Rachel Kearns, Rachel Lewis, Rana Madani, Ravi Moonka, Raza Sayyed, Rebecca Dawson, Rebecca Langley, Rebecca Logan, Richard Bamford, Richard Slater, Rob Stephens, Robert Arnott, Ross Kerridge, Ruth Parks, Ruth Quinn, Sahara Fleetwood-Beresford, Sally Laight, Sam Lovage, Samantha Black, Samantha de Silva, Samantha Hendren, Sarah Duff, Sarah Fitzgibbon, Sarah Grady, Sarah O’Farrell, Sarah Peacock, Sarah Russell, Sarah Squire, Sayuri Nakajima, Selina Ford, Semra Demirli, Setthasorn Zhi Yang Ooi, Shafaque Shaikh, Shana Hall, Shannon Knight, Shanthan Ganesh, Sharon Bassett, Sharon Hilton-Christie, Shirley Chan, Simon le Roux, Sonya McKinlay, Sophie Excell, Sophie Hamilton, Sreekrishna Kumar Ambalaparambil, Stacey Pickering, Steffen Seyfried, Stephen Chapman, Stephen Fenwick, Stephen O’Meara, Steve Harris, Stuart Armitage, Stuart Spear, Sue Hilsdon, Susan Chandler, Susannah Hill, Suzanne Rose, Terry Iddon, Theodore Liakakos, Thomas Pinkney, Usman Khan, Valerie Reid, Vardhini Vijay, Victoria Aubrey, Vidya Kasipandian, Viswanath Yks, Vlad Simianu, Wah Yang, William Ritchie, Yahya Aali, Yamin Bhat

**Affiliations:** Department of Surgery, The Christie NHS Foundation Trust, University of Manchester, Manchester, UK; Patient Representative, Liverpool, UK; Centre for Medical Informatics, Usher Institute, University of Edinburgh, Edinburgh, UK; Department of Surgery, Sheffield Teaching Hospitals NHS Foundation, University of Sheffield, Sheffield, UK; School of Cancer Sciences, Faculty of Medicine, University of Southampton, Southampton, UK; Perioperative and Critical Care Theme, NIHR Southampton Biomedical Research Centre, University Hospital Southampton/University of Southampton, Southampton, UK; The University of Edinburgh, Undergraduate Medical School, Edinburgh, UK; Departments of Surgery, Royal Alexandra Hospital, Paisley and Golden Jubilee National Hospital, Clydebank, and University of Glasgow, UK

**Key points**

Study defined core standards and core outcomes for prehabilitation for all types of colorectal surgery.
Co-produced by patients and healthcare professionals internationally.
Consensus achieved on 33 core standards (what prehabilitation should include, who should be offered prehabilitation and who should be part of the prehabilitation team) and 21 core outcomes.
The DiSCO core standards and core outcomes should be implemented into future colorectal prehabilitation research to achieve standardization, allow study comparison and expedite translation into patient care.

## Introduction

Elective colorectal surgery constitutes some of the most commonly performed operations worldwide^1,2^. Despite national databases reporting a low 90-day mortality rate (3–6%), postoperative morbidity is common and can delay in-hospital recovery, resulting in readmissions, reduced quality of life, and even reduce cancer-specific survival^1,3,4^.

Prehabilitation is the process of physical, nutritional and psychological optimization prior to surgery and can augment the successes reported by Enhanced Recovery After Surgery (ERAS) programmes^5–8^. Demonstrated as safe and feasible in colorectal patients, early trial data suggest that prehabilitation can reduce postoperative complications by 51%, as well as improving exercise capacity and decreasing length of hospital stay^9–13^.

To strengthen the evidence and expedite prehabilitation implementation, systematic reviews have combined the small number of trials, reporting that the heterogeneity of data limits comparison^12,14–16^. Limitations highlighted include: differing inclusion criteria focusing on patients with a malignant diagnosis and excluding those with benign pathology; differing methodology; variation in prehabilitation definition and disparity with the programme elements; and lastly, substantial variation in reported outcome measures. These reviews conclude that core standards and core outcome measures for prehabilitation are required. Core standards are a minimum set of agreed items that should be included in research methodology. Core outcomes are the minimum set of outcomes that should be reported in trials. Both core standards and core outcomes use relevant stakeholders, including patients, to achieve consensus and their subsequent adoption should improve the quality and comparison of future prehabilitation research^17–20^.

The aim of the DiSCO (Defining Standards in Colorectal Optimisation) study was to achieve international consensus from patients and healthcare professionals on core standards and core outcomes for clinical trials of prehabilitation in elective colorectal surgery.

## Methods

### Study overview

The methodology was adapted from the Core Outcome Measures in Effectiveness Trials (COMET) handbook and the recommended standards for core outcome set development^21^. As the aim was to develop a core set of standards and outcomes, the Core Outcomes Set–STandards for Development (COS-STAD) methodology was adapted^22^. Ethical approval was granted (University of Glasgow College of Medical, Veterinary and Life Sciences Ethics Committee; 200190120). The study was registered with COMET Initiative (https://www.comet-initiative.org/Studies/Details/1716).

Core standards and outcomes were developed in three stages: long listing of standards and outcomes from systematic review and supplemented by a patient and public involvement (PPI) day; two rounds of Delphi process (2021–2022); and two consensus meetings to review Delphi survey results (4th and 5th March 2022). The protocol for the study has previously been published^23^.

### Scope

In line with the COS-STAD recommendations, the intended use of the core standards and outcomes (setting) is for research and clinical practice; the health condition was colorectal disease, population was adults ≥18 years old, and the intervention was prehabilitation prior to surgery. Colorectal disease was defined as any benign or malignant colorectal conditions treated with elective resection of part/all of the colon, rectum or anus. These conditions included but were not limited to colorectal cancer, anal cancer, diverticulitis, inflammatory bowel disease and pelvic floor dysfunction.

### Steering group and stakeholders

To ensure inclusivity and diversity of potential stakeholders and participants, leading national and international professional bodies in colorectal disease and/or those endorsing prehabilitation and/or components of prehabilitation were identified and approached (*Appendix 1*). For healthcare professionals (HCPs) this would include the following specialties: colorectal surgeons, colorectal anaesthetists, colorectal nurse specialists, colorectal oncologist (medical or clinical), exercise oncologists, exercise physiologists, sports scientists, sports medicine specialists, physical exercise/activity specialists, nutritionists or dieticians, psychologists, geriatricians, pharmacists and general practitioners. An international steering group (UK, USA, Canada, New Zealand, Australia, Europe) was set up to identify these professional bodies and to ensure widespread distribution of the Delphi survey and consensus days through social media (@DiSCO_study). This work was co-produced with patients, evidenced by a patient research partner as a lead member of the steering group and the inclusion of patient-centred professional groups and charities as stakeholders/participants.

### Stage 1: long-listing

The long list of standards and outcomes was extracted from the systematic review on prehabilitation performed by the DiSCO study team^14^. Briefly, from 33 studies with a total of 3962 patients, the DiSCO steering group analysed their methodology and primary and secondary outcomes to develop a list of items that could be considered for inclusion. A PPI day was undertaken to ensure that the wording and meaning of the long-listed standards was clear and that terminology was understandable. Discussion also focused on how patients and families could be impacted by certain elements of prehabilitation, and the feedback from this discussion was used by the steering group to inform rationalization of the long list into question items for the Delphi questionnaire and by the chair to guide discussion at the consensus meetings.

The final long list of standards and outcomes were reviewed by the steering group for definition, duplication, clarity and for plain English, and used to populate the Delphi questionnaire with clear definitions and plain language descriptions accompanying each item (*Appendix 2*).

### Stage 2: Delphi survey

A two-round modified Delphi questionnaire was conducted (DelphiManager platform) and participants registered online via the COMET Delphi Manager. The registration process included participant consent, and captured name, email, stakeholder group (patient or HCP) and country of residence.

During each round, participants were asked to rate the importance of each of the items using the Likert scale from 1 (not important) to 9 (critically important): 1–3 signifies the item is of little importance, 4–6 some importance and 7–9 critical importance^22^. At the end of round 1, participants were invited to suggest any additional items for inclusion in round 2. These additional items were discussed at a steering group meeting and those deemed relevant by the majority were taken into round 2. Participants who completed round 1 were sent an email invitation to participate in round 2, followed by one reminder. In round 2, participants reviewed the scores they had given items in round 1 alongside the summarized scores of other participants (average score for each item presented as histograms) stratified by stakeholder group, before rescoring each item.

#### Consensus criteria

To reduce bias, predetermined consensus thresholds were used: items ranked as of critical importance (7–9) by ≥70% and of little importance (1–3) by ≤15% of participants in both stakeholder groups were categorized as ‘consensus-in’. Items ranked as of critical importance (7–9) by ≤50% or of little importance (1–3) by ≥50% participants in both stakeholder groups were categorized as ‘consensus-out’. Any items not reaching either the threshold for ‘consensus-in’ or ‘consensus-out’ were considered ‘borderline’ (*Table 1*).

**Table 1:** Consensus criteria for Delphi questionnaire and consensus meeting

Percentage of participants scores		Patients			
		≥70% 7–9 and <15% 1–3	50–70% 7–9	<50% 7–9	≥50% 1–3
Healthcare professional	≥70% 7–9 and <15% 1–3	Consensus-in	Borderline	Borderline	Borderline
	50–70% 7–9	Borderline	Borderline	Borderline	Borderline
	<50% 7–9	Borderline	Borderline	Consensus-out	Consensus-out
	≥50% 1–3	Borderline	Borderline	Consensus-out	Consensus-out

Items meeting the criteria for ‘consensus-in’ after round 1 of the Delphi were directly added to the final shortlist and not included in subsequent rounds. All other items (consensus-out and borderline) were taken forward to round 2. After round 2, any additional items reaching the threshold for ‘consensus-in’ were directly added to the shortlist. Any items ranked ‘consensus-out’ were excluded. All borderline outcomes were taken forward for discussion at the consensus meeting.

### Protocol deviation

Following round 2, 53 items had already achieved the predefined threshold for consensus and the steering group agreed that there was little additional benefit in asking participants to complete the planned third round of the Delphi and risk further attrition of participants through questionnaire fatigue.

### Stage 3: consensus meeting

Due to COVID restrictions and to allow international participation, two online consensus meetings were planned (one for core standards, one for core outcomes) and held on consecutive days, at different times, for 3 h each. Previous DiSCO participants were invited with additional participants recruited via X (formerly Twitter) and direct e-mail. Purposive sampling of potential participants was undertaken to ensure a wide a range of geographic and stakeholder representation. Voting during the consensus meeting was conducted using Mentimeter online voting software (www.mentimeter.com), allowing electronic consent for participation to be taken. Participants were asked to select their stakeholder group (patient or HCP). The meeting was co-chaired by members of the steering group (R.F.: experienced consensus meeting facilitator and core outcome set methodologist; S.B.: patient experienced in health consensus meetings). The meeting summarized the aims of the project and the items that had achieved consensus with no objections raised. Borderline items were discussed and voted on. Stakeholder stratification of voting results was displayed as an average score for each item, presented as histograms. The criteria for consensus were the same as for the Delphi survey. Results were displayed immediately after voting for each item. The meeting concluded with the final core standards and outcomes set displayed and ratified.

## Results

### Long-listing

**Fig. 1:**
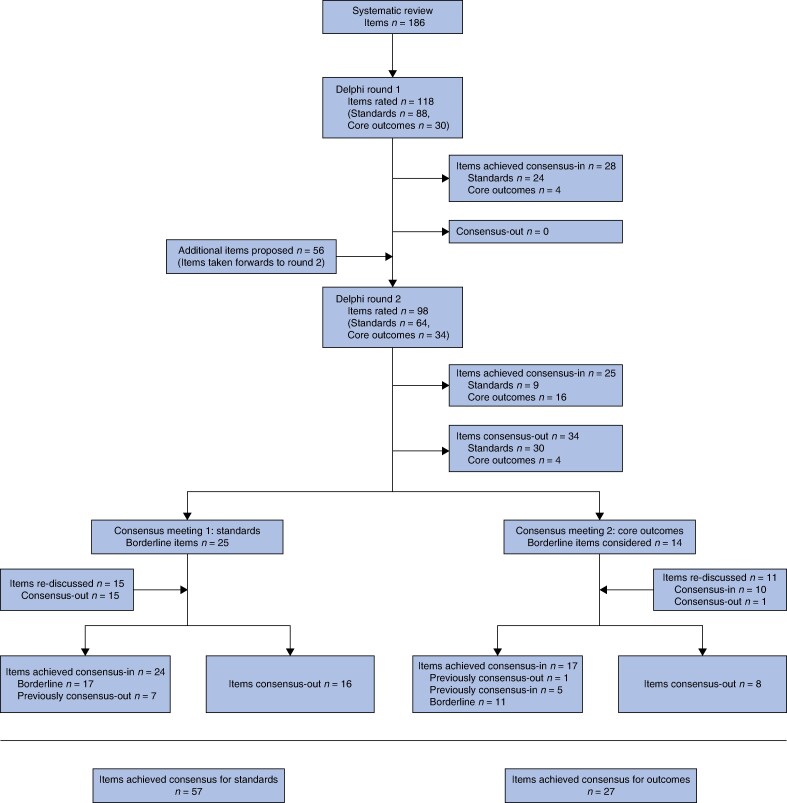
Flow of items of standards and core outcomes through DiSCO Delphi

The systematic review identified 186 items—standards and outcomes^14^ (*Fig. 1*). After merging closely related items and excluding items that were clinically inappropriate or out of context, the steering group proposed a final long list of 118 items across nine domains: components of prehabilitation, setting of prehabilitation, exercise/physical activity, nutrition, psychological support, comprehensive geriatric assessment, recipients of prehabilitation, delivery of prehabilitation and outcomes (*Appendix 2*).

### Delphi survey

In total, 289 participants from 18 countries registered for round 1: 51 patients and 238 HCPs. Of the 289, 8 participants did not answer any questions (4 HCPs and 4 patients) and 233 participants (198 HCPs and 35 patients) answered all questions. Participant characteristics from each round are in *Table 2*. Round 1 was open for 10 weeks, extended from 6 to 7 weeks to maximize participant numbers and accommodate a holiday period.

**Table 2:** Participant characteristics for prehabilitation in colorectal surgery Delphi

	Delphi		Consensus meetings	
	Round 1	Round 2	Standards	Outcomes
**Participants**				
Patient	51	30	9	6
Healthcare professionals	236	163	25	20
Anaesthetist	24	17	4	2
Exercise specialist	6	5	1	0
Exercise physiologist/sports scientist	5	4	0	1
General practitioner	2	2	0	0
Geriatrician	8	3	1	0
Nutritionalist/dietician	29	17	4	2
Oncologist	1	1	0	0
Physiotherapist	16	11	2	2
Psychologist	1	1	0	0
Specialist nurse	22	11	1	2
Surgeon	110	81	9	8
Unknown	0	0	3	3

**Country of residence/practice**	**Rounds 1 and 2**		
Europe	Austria 1 Finland 1 France 1 Germany 1 Greece 1 Ireland 7 Italy 3 Spain 2 Sweden 2 Turkey 2 UK 219	Italy 1 Ireland 2 UK 23	Italy 1 UK 20
North America	Canada 4 USA 13	Canada 4	Canada 1 USA 1
Australasia	Australia 19 New Zealand 4	0	Australia 1 New Zealand 1
Asia	China 1 India 1	China 1 India 1	Japan 1
Other	6	2	0

After round 1, 28 items achieved consensus-in (*Fig. 1*, *Table S1*). Participants proposed 56 additional items (*Appendix 3*). After steering group review, eight items were included with the rest excluded as either already included, or not within the scope of the study (being neither a standard nor outcome). Ninety-eight items were taken forward to round 2.

Round 2 was open for 7 weeks. All questions were answered by 186 people (156 HCPs, 30 patients). After round 2, 25 items achieved consensus-in and 34 consensus-out. A total of 39 items meeting the criteria for ‘borderline’ were taken forward to the consensus meetings.

Excluding participants who registered but did not answer any questions, attrition from round 1 to round 2 was 34% (HCPs 34%, patients 36%). Among participants who answered all questions in round 1, attrition was 20.1% (HCP 21%, patients 14%).

### Consensus meetings

The standards and outcome consensus days were attended by 34 (25 HCP, 9 patients) and 26 (20 HCP, 6 patients) participants respectively (*Table 2*).

#### Core standards consensus meeting

At the core standards consensus meeting, 25 borderline items spanning five domains were considered: setting for prehabilitation, exercise/physical activity, nutrition, psychological support and who should deliver prehabilitation. The standards in the remaining domains had already achieved consensus. The steering group proposed rediscussion of 15 items that were consensus-out in the Delphi but were closely related to borderline items that were being discussed, resulting in a total of 40 items considered. Description of the discussions that took place around core standards are provided in *Appendix 4*.

In total, 57 items relating to core standards met the threshold for consensus-in after the Delphi and consensus meetings. Reconciliation of items with multiple options resulted in a final list of 33 core standards. The final list of core standards was presented and agreed by all participants at the end of the consensus meeting (*Table S1* and *Table 3*).

**Table 3:** Final set of core standards for prehabilitation research in colorectal surgery

Domain	Subdomain	Standards (*n* = 33)
Components of prehabilitation		Exercise
		Nutrition
		Psychological (emotional) support
		Comprehensive geriatric assessment (for older, frail patients)
Setting for prehabilitation		Multicentre options
Exercise/physical activity	Medium	Choice of face-to-face or remote
	Group size	Choice of one-to-one or group
	Personalization	A personalized exercise programme specifically tailored to the individual
	Type	Functional activity training
		Cardiovascular/aerobic exercise
	Duration	The exercise programme should last 2–4 weeks
Nutrition	Medium	Choice of face-to-face or remote
	Group size	One-to-one nutritional advice
	Personalization	A personalized nutritional advice programme specifically tailored to the individual
	Duration	The nutrition programme should last 4–6 weeks
Psychological support	Medium	Choice of face-to-face or remote
	Group size	One-to-one psychological support
	Personalization	A personalized psychological support programme specifically tailored to the individual
	Type	Focus on anxiety reduction
		Focus on body image including stoma concerns
		Relaxation techniques (e.g. breathing exercises, yoga)
		Mental preparedness and motivation
	Duration	Psychological support should last 2–6 weeks
Comprehensive geriatric assessment		All components of the comprehensive geriatric assessments
Recipients of prehabilitation	Reason for surgery	All types of colorectal surgery for any condition, including patients having neoadjuvant chemotherapy
	Age	Patients of any age
	Co-morbidities and risk factors	Patients with any co-morbidities and additional risk factors
Delivery of prehabilitation		Specialist nurse
		Exercise physiologist or sports scientist
		Physiotherapist
		Nutritionist/dietician
		Psychologist
		Other patients who are having/have had colorectal surgery

#### Core outcomes consensus meeting

The steering group proposed grouping items into six domains based on the recommended outcome taxonomy from the COMET initiative^24^: physiological/clinical, life impact, global quality of life and well-being, adverse events, death and resource use. The domain allocation of the 16 items that had achieved consensus-in was agreed by participants. Ten outcomes that had met the criteria for consensus-in and one that met the criteria for consensus-out were reintroduced due to potential overlap with the 14 borderline outcomes as outlined below. The result for every individual outcome at every stage of the consensus process, including how items were combined, is shown in *Table S1*.

Two items reintroduced for discussion were overall quality of life and overall health and well-being. Both had achieved consensus-in and were considered for merging into one item called ‘global quality of life and well-being’. Terminology was explained and discussion facilitated. Participants felt that these items addressed sufficiently different concepts and voted to keep them as two separate items. Description of the discussions that took place around outcomes are provided in *Appendix 5*.

In total, 27 items relating to core outcomes met the threshold for consensus-in after the Delphi questionnaire and consensus meetings. After merging items relating to measures of physical or cardiorespiratory function had been agreed as described above, 21 core outcomes across six domains were agreed for future prehabilitation research (*Table 4*). The final list of core outcomes, including all the proposed merging/reconciliation of items, was presented and agreed by all participants at the end of the consensus meeting.

**Table 4:** Final set of core outcomes for prehabilitation research in colorectal surgery

Domain	Subdomain	Core outcome set (*n* = 21)
Physiological/clinical	Musculoskeletal	A suitable objective measure of physical function
	Cardiorespiratory	A suitable physiological measure of cardiorespiratory fitness
	Metabolism and nutrition	Nutritional assessment
	General	Pain
	Neoplastic	Relevant condition-specific outcomes with reference to the relevant core outcome set where available
Life impact	Physical function	Sleep
		Bowel function
		Return to normal physical activities
		Fatigue
	Psychiatric/emotional functioning	Cognitive issues
		Anxiety
		Depression
		Stoma concerns
		Stress
	Behavioural	Patient activation measures
Global quality-of-life and well-being		Overall quality of life
		An overall measure of health and functioning
Adverse events	Adverse events	Relevant condition-specific outcomes with reference to the relevant core outcome set where available
Death	Survival	Survival
Resource use	Societal/carer burden	Discharge destination and support requirements
		Family/carer involvement

## Discussion

This international consensus work including healthcare professionals and patients provides consensus on core standards and core outcomes for future clinical trials for prehabilitation in colorectal surgery. Prehabilitation research is a rapidly evolving area and the recent international agreement on the top 10 research priorities in prehabilitation makes publication of these core standards and core outcomes timely^25^. The robust process applied should allow implementation to be widely acceptable across a range of healthcare systems and health specialties. Standardization should improve between-study comparisons and accelerate knowledge about prehabilitation in the care of colorectal surgical patients.

Future prehabilitation research should consider these core standards and core outcomes to be the minimum standards and outcomes to be included. Importantly, this work provides a flexible framework where other relevant published core standards and core outcomes can be included^26–28^. Researchers can use both sets together, but they can also be applied individually depending on the research design.

The aim to co-produce with patients was achieved with a patient research partner as a lead investigator, a dedicated PPI event and engagement of patients and patient groups through each step of the Delphi process and consensus meetings. Consideration for the multiple stakeholders involved in colorectal prehabilitation research was paramount with approaches for recruitment through prehabilitation and colorectal specialties’ professional bodies. The DiSCO Delphi process and consensus meeting brought these stakeholders together for the first time using best-practice methodology, including question order randomization and displaying feedback stratified by stakeholder group between rounds. Another strength is the inclusion of benign colorectal conditions, as this population is often overlooked in favour of malignant disease in prehabilitation research.

In common with many consensus studies, it is likely that recruitment bias is present. Individuals who did not feel willing or able to participate might differ in opinions from those who did participate. Combining core standards and core outcomes resulted in a lengthy long list that may account for the attrition rate between rounds. The steering group initially considered focusing on core outcomes only, but the strong interplay between the standards and outcomes meant that it was felt important to include both. The recruitment strategy using both social media and direct approaches to relevant international professional bodies may not have reached all potential contributors who may have wanted to participate. Finally, there was a predominance of participants from European countries with very few low-income countries. This reflects the distribution of published prehabilitation research worldwide, highlighting the need for future research in the low-income country setting.

Implementation of core standards and core outcomes for prehabilitation research in colorectal surgery should enable progression to a large body of research that in addition to enabling high-quality meta-analyses will ensure surgeons and prehabilitation specialties communicate using the same language^29^. This is key due to the substantial range of stakeholders in prehabilitation research. Trial management groups of current prehabilitation studies should review DiSCO and consider implementing the core standards and core outcomes. Future work could entail the development of a core measurement set to achieve the core standards and core outcomes defined here. Consideration will need to be given to individual needs and feasibility, in addition to the range of prehabilitation interventions that could be performed. Using the physiological core outcomes as an example, this could include anaerobic threshold testing, aerobic testing or strength testing.

The DiSCO core standards and outcomes represent the consensus opinion of international stakeholders involved in prehabilitation research in colorectal surgery. Implementation of the DiSCO core standards and core outcomes for current and future trials will create a common language that should facilitate comparative evidence synthesis, thereby accelerating translation of prehabilitation research into patient benefit.

## Author contributions

S.M., S.B., and R.F. planned and designed the study. Systematic review was led by S.D. PPI day developed and run by S.M., I.P. and S.B. Long listing was performed by the steering team, S.M., S.B., R.F., S.D., M.W. R.F. set up and administered the Delphi survey. S.M. set up the stakeholders list and contacts. R.F. and S.R.K. analysed the Delphi results and structured the consensus days. In addition to R.F., S.B. and S.R.K. facilitated the consensus meetings, with assistance provided by C.B., J.N., M.T. The steering group produced the first written draft with all authors approached for revision and subsequent agreement of the final draft.

Sue Blackwell (Conceptualization, Data curation, Methodology, Writing—original draft, Writing—review & editing), Rebecca Fish (Conceptualization, Data curation, Formal analysis, Methodology, Writing—original draft, Writing—review & editing), Stephen Knight (Data curation, Formal analysis, Methodology, Writing—original draft, Writing—review & editing), Sarah Daniels (Methodology, Writing—original draft, Writing—review & editing), Malcolm West (Methodology, Writing—original draft, Writing—review & editing), Iona Pearson (Methodology, Writing—original draft, Writing—review & editing), and Susan Moug (Conceptualization, Formal analysis, Funding acquisition, Methodology, Supervision, Writing—original draft, Writing—review & editing)

## Collaborators

Deborah S. Keller, Nanette Mutrie, Paul Kelly, Nicola S. Fearnhead, Carly Bisset, Jene Ng, May Thu, Mike Kelly. Adam Boutall, Aileen Schofield, Alastair McKay, Alec Mcdonald, Alex Labey, Alexander Heriot, Alexandros Charalabopoulos, Alice Finch, Alison Culkin, Alison Payne, Alistair Owen, Amanda Reid, Amy Kerr, Andrei Tanase, Andrew Dennis, Andrew Miller, Andrew Renehen, Andrew Renwick, Andrew Rogerson, Andriana Petrova, Angela Baker, Angeline Adams, Angeline Price, Angie Balfour, Anisa Kushairi, Ann-Maree Murphy, Anne Marks, Annelies Mittendorff, Annie McCaffery, Arman Erkan, Arnold Goede, Ash Bhalla, Asif Mehraj, Ayse Gizem Unal, Baljit Singh, Ben Griffiths, Beryl Goddard, Bill Campbell, Brian Devlin, Brian Power, Cain Clark, Caroline Dodds, Carolyn Atkin, Catherine Radley, Catriona Brown, Charles Hendrickse, Charlotte Atkinson, Charlotte Foley, Chelsia Gills, Chloe Beard, Chloe M Grimmett, Chloe Nottingham, Christopher J. Smart, Claire Cruikshanks, Claire Finlayson, Claire Hall, Claire Knowles, Claire McCann, Claire Taylor, Clare E. Collins, Clare Shaw, Colette Backwell, Craig Gilmour, Craig Lynch, Danella Ruddy, Daniel Santa Mina, Dave Pawson, David Easton, David Lubowski, David Oglesby, David Proud, David Shipway, David Watt, David Yates, Dean Harris, Debbie Mulhern, Deborah Howland, Deborah Keller, Deepa Muthukrishnan, Denny Levett, Dermot Burke, Duncan Miller, Effie Jamieson, Efstratia Baili, Eleanor Gray, Eleanor Hitchman, Eleftheria Douka, Emma Greenwood, Emma McMillan, Emma Nicholls, Evgeniy Drozdov, Fergal Fleming, Fiona Windsor, Fionna Martin, Francesco Maria Carrano, Franco Carli, Fraser Smith, Gary Howell, Gary Nicholson, Geert Koffeman, Gemma Faulkner, Gemma Ford, Georgina Giebner, Gianluca Pellino, Glen Guerra, Gregory Thomas, Heather Gilmore, Heather MacKinnon, Honor Blackwood, Hugh Paterson, Hwei Jene Ng, Hytham K. S. Hamid, Iain Jourdan, Ian Bissett, Ian Daniels, Isla Veal, Jackie Timperley, James Dilley, James Glasbey, James Holding, James Toh, Jamie Alcock, Jan Clarke, Jane Booker, Javier Ripollés-Melchor, Jeanette Osborne, Jeanette Preston, Jennie Burch, Jennifer Edwards, Jennifer Mackney, Jennifer Stewart, Jennifer Henderson, Jenny Pipe, Jenny Woodward, Jeremy Williamson, Jessica Bower, Jessica Mijnssen, Jo Lloyd, Joanna Flint, Joanne Logan, John Jameson, John Woodfield, Jon Lund, Jonathan Heath, John Moore, Jugdeep Dhesi, Julie Berson, Julie Wilkinson, June Davis, Justin Davies, Karen Kerr, Karen O’Hare, Karen Robb, Karen Telford, Karina Va Zquez-Narvaez, Karol Pal, Kathy Borthwick, Kausik Ray, Kellie Owen, Kenny Nattrass, Kerry S. Courneya, Khalid Osman, Kimberley Adams, Kirsten Cassidy, Kirsty Rowlinson Groves, Kirsty Wade, Krishanthi Sathanandan, Krishna Kholia, Laura Hancock, Laura McGarrity, Lauren Coyle, Leah Cox, Lena W. S. Ngu, Leon Fu, Liam Humphreys, Linda Tutty, Liz Murphy, Lorraine Hughes, Louise Hunt, Louise Maxwell, Louise Perryman, Loukas Nadiotis, Luke Wheldon, Manisha Shah, Margaret Clark, Maria Burton, Marie Sheahan, Mark Bagnall, Mark Graham, Martin Rutegard, Mary Schactler, Marylise Boutros, Matthew J. Lee, Mhairi Burke, Mhairi Simpson, Michael Davies, Michael P. Kelly, Michael Lim, Michael Suen, Michele Carvello, Michelle Willcocks, Mike Grocott, Mohamed Shams, Monica Millan, Mukul Dube, Nadine Harran, Natalie Smith, Andrew Renehan, Omer Aziz, Nauman Ahmed, Neil Agnew, Neil Bibby, Neil Smart, Nicola Dames, Nicola Hill, Nicola Maguire, Nicola Peat, Nicole Saur, Nigel Horwood, Nigel Richardson, Nurulamin Noor, Peter Ishak, Peter Loder, Peter Murchie, Philip Walton, Pia Bernardi, Rachael Clifford, Rachel Hargest, Rachel Kearns, Rachel Lewis, Rana Madani, Ravi Moonka, Raza Sayyed, Rebecca Dawson, Rebecca Langley, Rebecca Logan, Richard Bamford, Richard Slater, Rob Stephens, Robert Arnott, Ross Kerridge, Ruth Parks, Ruth Quinn, Sahara Fleetwood-Beresford, Sally Laight, Sam Lovage, Samantha Black, Samantha de Silva, Samantha Hendren, Sarah Duff, Sarah Fitzgibbon, Sarah Grady, Sarah O’Farrell, Sarah Peacock, Sarah Russell, Sarah Squire, Sayuri Nakajima, Selina Ford, Semra Demirli, Setthasorn Zhi Yang Ooi, Shafaque Shaikh, Shana Hall, Shannon Knight, Shanthan Ganesh, Sharon Bassett, Sharon Hilton-Christie, Shirley Chan, Simon le Roux, Sonya McKinlay, Sophie Excell, Sophie Hamilton, Sreekrishna Kumar Ambalaparambil, Stacey Pickering, Steffen Seyfried, Stephen Chapman, Stephen Fenwick, Stephen O’Meara, Steve Harris, Stuart Armitage, Stuart Spear, Sue Hilsdon, Susan Chandler, Susannah Hill, Suzanne Rose, Terry Iddon, Theodore Liakakos, Thomas Pinkney, Usman Khan, Valerie Reid, Vardhini Vijay, Victoria Aubrey, Vidya Kasipandian, Viswanath Yks, Vlad Simianu, Wah Yang, William Ritchie, Yahya Aali, Yamin Bhat.

## Supplementary Material

znae056_Supplementary_Data

## Data Availability

All relevant data are presented in the article. No further data are available.

## Appendices

**Appendix 1 List of participating professional and charitable bodies for prehabilitation in colorectal surgery Delphi**

**Table:**

Professional body
International Society of Behavioural Nutrition and Activity
Scottish Physical Activity Research Connections
Scottish Cancer Prevention Network
The Association of Surgeons of Great Britain and Ireland
The Association of Coloproctology of Great Britain and Ireland
Royal College of Anaesthetists and TriPom—Trainees with an interest in perioperative medicine
National Enhanced Recovery after Colorectal Surgery Initiative)
MacMillan Cancer Support
Bowel Cancer UK
Crohn's and Colitis UK
Ileostomy Association UK
Colostomy UK
Colorectal Surgical Society of Australia and New Zealand
ERAS plus, Manchester, UK
European Society of Coloproctology
American Society of Colorectal Surgeons

**Appendix 2 Long list of items (*n* = 118) and associated domains for prehabilitation in colorectal surgery Delphi**

**Table:**

Outcome/standard name	Domain no.	Outcome ID
Exercise	1	1
Nutrition	1	2
Psychological (emotional) support	1	3
Comprehensive geriatric assessment (for older; frail patients)	1	4
In secondary care (the hospital)	2	5
In primary care (the GP’s practice)	2	6
In the community; for example, at a local gym or community centre	2	7
Face-to-face exercise supervision and advice	3	8
Remote exercise supervision and advice (e.g. by telephone or video-call)	3	9
One-to-one exercise supervision and advice	3	10
Group exercise supervision and advice	3	11
A personalized exercise programme specifically tailored to the individual	3	12
A standardized exercise programme designed for prehab but not specifically tailored to each individual	3	13
General exercise advice not specifically designed for prehabilitation	3	14
Exercise that becomes progressively harder	3	15
High-intensity/interval training	3	16
Endurance	3	17
Pulmonary physiotherapy exercises	3	18
Functional activity training	3	19
Cardiovascular/aerobic exercise	3	20
Resistance/weight training	3	21
Stretching/flexibility exercise	3	22
The exercise programme should last up to 2 weeks	3	23
The exercise programme should last 2–4 weeks	3	24
The exercise programme should last 4–6 weeks	3	25
The exercise programme should be in excess of 6 weeks	3	26
Face-to-face nutritional advice	4	27
Remote nutritional advice (e.g. by telephone or video-call)	4	28
One-to-one nutritional advice	4	29
Group nutritional advice	4	30
A personalized nutritional advice programme specifically tailored to the individual	4	31
A standardized nutritional advice programme designed for prehabilitation but not specifically tailored to the individual	4	32
General nutritional advice	4	33
The nutrition programme should last up to 2 weeks	4	34
The nutrition programme should last 2–4 weeks	4	35
The nutrition programme should last 4–6 weeks	4	36
The nutrition programme should be in excess of 6 weeks	4	37
Face-to-face psychological support	5	38
Remote psychological support (e.g. by telephone or video-call)	5	39
One-to-one psychological support	5	40
Group psychological support	5	41
A personalized psychological support programme specifically tailored to the individual	5	42
A standardized psychological support programme designed for prehabilitation but not specifically tailored to the individual	5	43
General advice on psychological support	5	44
Focus on anxiety reduction	5	45
Focus on body image including stoma concerns	5	46
Relaxation techniques (e.g. breathing exercises; yoga)	5	47
Mental preparedness and motivation	5	48
The psychological support should last up to 2 weeks	5	49
The psychological support should last 2–4 weeks	5	50
The psychological support should last 4–6 weeks	5	51
The psychological support should be in excess of 6 weeks	5	52
Cognitive assessments	6	53
Medication optimization	6	54
Co-morbidity review	6	55
Falls advice	6	56
Advanced care planning	6	57
Patients undergoing surgery for benign conditions	7	58
Patients undergoing surgery for cancer	7	59
Patients undergoing laparoscopic (keyhole) surgery	7	60
Patients undergoing open surgery	7	61
Patients undergoing chemotherapy or radiotherapy prior to surgery	7	62
Patients having a stoma formed as part of surgery	7	63
Patients under 60 years of age	7	64
Patients aged 60–69	7	65
Patients aged 70–79	7	66
Patients aged 80–89	7	67
Patients aged 90 and over	7	68
Frail patients	7	69
High-risk patients	7	70
Malnourished/underweight patients	7	71
Obese patients	7	72
Patients with recent or long-term mental illness	7	73
Surgeon	8	74
Anaesthetist	8	75
Specialist nurse	8	76
Oncologist (medical or clinical)	8	77
Exercise physiologist or sports scientist	8	78
Exercise oncologist	8	79
Sports medicine specialist	8	80
Exercise/activity specialist (e.g. a personal trainer)	8	81
Physiotherapist	8	82
Nutritionist/dietician	8	83
Geriatrician	8	84
Pharmacist	8	85
Psychologist	8	86
General practitioner	8	87
Other patients who are having/have had colorectal surgery	8	88
Daily or weekly step count	9	89
Cardiopulmonary exercise test	9	90
Sit-to-stand	9	91
6-min walk test	9	92
Respiratory/breathing measurements (e.g. peak flow)	9	93
Adherence to rehabilitation (e.g. number of exercise sessions completed)	9	94
Handgrip strength	9	95
Leg strength (e.g. leg/quadriceps extension)	9	96
Percentage body fat	9	97
Weight change	9	98
Energy expenditure	9	99
Change in nutritional assessment	9	100
Fatigue	9	101
Anxiety	9	102
Depression	9	103
Stoma concerns	9	104
Stress	9	105
Sleep	9	106
Pain	9	107
Bowel function	9	108
Overall quality of life	9	109
Return to normal activities	9	110
Cognitive issues	9	111
Length of hospital stay	9	112
Complications	9	113
Length of critical care stay (high-dependency unit or intensive care)	9	114
Discharge destination and support requirements	9	115
Inability to complete physical tests	9	116
Planned surgery does not go ahead	9	117
Prehabilitation stopped	9	118

Domains: (1) components of prehabilitation; (2) setting of prehabilitation; (3) exercise/physical activity; (4) nutrition; (5) psychological support; (6) comprehensive geriatric assessment (7); recipients; (8) delivery; (9) outcomes.

**Appendix 3 Review of 56 additional items proposed by participants after round 1 of the DiSCO study**

**Table:**

Outcome	Score	Is it an outcome/standard? (yes/no)	Is it already included? (yes/no)	Matched outcome if already included (outcome ID)	Final decision	Comments
To measure what benefit the prehabilitation has on the patient's recovery if serious complications occur. Such as are they off the ventilators earlier; ability to walk independently sooner; are they coping better psychologically, etc.	9	Yes	Yes	112, 113, 109	Already included—no further action required	
Body composition—muscle mass/muscle quality	8	Yes	No	96, 97, 98	Already included—modification to existing outcome/standard wording needed to clarify	
My support network, a.k.a. my family and friends	9	No	No		Not an outcome/standard	
Prehabilitation during COVID	9	No	No		Not an outcome/standard	
How to manage patient expectations	9	No	No		Not an outcome/standard	
Healthcare professionals’ likelihood to adapt to a patient’s personal nutritional and physical therapy when those standards are not within protocols—computer says no	9	No	No		Not an outcome/standard	
Allow me as the patient to document the success of the intervention pre, during and post. I would like it above my bed ‘prehab optimized and independent documenter’, ha ha what are the chances?	9	No	No		Not an outcome/standard	
Abdominal muscle function/activation measured by ultrasound	9					
Preop input is so vital in getting the best post surgery. The psychological side of surgery coupled with the need to look at diet should be paramount for the best possible recovery. Using veteran patients to support existing patients along with the expertise of a dietician and colorectal doctor or nurse would only aid a speedier recovery; when patients get left behind so does their morale and subsequent recovery time	9	Yes	Yes	88	Already included—no further action required	
Pulse wave velocity (a measure of vascular stiffness)	8	Yes	No		To be added to round 2	
Patient’s spouse should be involved in the whole prehabilitation process (offered/delivered)	9	Yes	No		To be added to round 2	
% muscle mass	6	Yes	No	96, 97, 98	Already included—modification to existing outcome/standard wording needed to clarify	
Survival outcomes	7	Yes	No		To be added to round 2	
Quality of life scores	9	Yes	Yes	109,110	Already included- no further action required	
Completion of chemotherapy/radiotherapy	7	Yes	No	117	Already included—modification to existing outcome/standard wording needed to clarify	
Patient experience	9	yes	yes	109	Already included—no further action required	
WHODAS 2.0 (assessment of health and disability)	8	Yes	Yes	90–99 and 109–111	To be added to round 2	
Short physical performance battery	7	No	No	90–99 and 109–111	Not an outcome/standard	
Family/carer voice	9	Yes	No		To be added to round 2	
Prehab should be community-based with leverage into long-term exercise behaviour change	9	Yes	Yes	8 to 11	Already included–no further action required	
Compliance with postop ERAS goals	7	No	No		Not an outcome/standard	
Cancer recurrence rate	6	Yes	No		To be added to round 2	
Cost saving of prehabilitation programme	7	No	No		Not an outcome/standard	
Patient activation measures	8	Yes	Yes	109, 110	To be added to round 2	
Joining a peer group for support from other similar patients with more experience	6	No	No		Not an outcome/standard	
Mentoring with one-on-one contact to another patient in similar situation	5	Yes	Yes		Already included—no further action required	
Prehabilitation for friend or family member who will support the patient's recovery at home	7	No	No		Not an outcome/standard	
Are patients expectations met?	6	No	No		Not an outcome/standard	
How important is the role of local cancer support charities in signposting to prehabilitation advice?	7	No	No		Not an outcome/standard	
How important is role of community-based charities and other support groups in delivery of prehabilitation?	7	No	No		Not an outcome/standard	
Risk triage tool that medically and rehabilitaion dichotomizes prehabilitation assessment and intervention needs to support programme	9	No	No		Not an outcome/standard	
Changes in negative lifestyle behaviours (e.g. smoking; drinking >14 units alcohol per week; amount of physical activity per week) (WHO guidelines)	9	Yes	No		To be added to round 2	
DASI score	7	Yes	Yes		To be added to round 2	Think this and WHODAS can be added as a separate item ‘global measure of health and function, e.g. WHODAS or DASI score’
Qualitative analysis of prehabilitation (e.g. acceptance to patients and healthcare professionals)	9	No	No		Not an outcome/standard	
Patient activation measure as measure of patient self-efficacy—important to commissioners	7	Yes	Yes	109, 110	To be added to round 2	I think this is different—needs adding as its own item
Postoperative course longer than hospital stay (e.g. A&E attendances; readmission rates and primary care visits up to 12 months post-surgery)	7	Yes	No		To be added to round 2	
Vitamin D assessment	9	No	No		Not an outcome/standard	
Institution-free days to 12 months after surgery	9	No	No		Not an outcome/standard	
Consultant needs to encourage prehabilitation in the initial instance to the patient	9	No	No		Not an outcome/standard	
Specialist nurse needs to encourage prehabilitation to the patient	9	No	No		Not an outcome/standard	
Contact with the physiotherapist (face to face—if possible)	9	Yes	Yes	82	Already included- no further action required	
Cardiopulmonary exercise test	9	Yes	Yes	90	Already included—no further action required	
Assessment with the physiotherapist to identify objectives and plan of prehabilitation	9	Yes	Yes	82	Already included—no further action required	
Outcome measures—6MWT; sit to stand; grip strength; BMI; maximum inspiratory pressure (MIP); balance test	9	Yes	Yes	89–99	Already included—no further action required	
Food diary given to patient in initial assessment with the physio then after this has been kept a few days—a dietetics assessment	9	Yes	Yes	27–33	Already included—no further action required	
Bespoke gym programme with a gym instructor/exercise physio—overseen by the physio 3–4× weekly—supervised	9	Yes	Yes	23–26	Already included—no further action required	
Inspiratory muscle training 2× daily—supervised if possible/or via telephone with patients keeping a record of their progress for feedback	9	Yes	Yes	18	Already included—no further action required	
Physio can flag up potential function needs if they need and possible things they’ll require from a psychologist	9	No	No		Not an outcome/standard	
Training programme should be completed for at least 4 weeks prior to surgery but we’ve seen positive outcomes with only 2 weeks training	9	Yes	Yes	23–26	Already included- no further action required	
Re-do outcomes the week before their surgery	9	No	No		Not an outcome/standard	
Physio throughout can manage patient expectations and what will be expected of them the day post-surgery (i.e. getting out of bed)	9	No	No		Not an outcome/standard	
Physiotherapist who prehabbed the patients sees the patient the day post-surgery as they will know their baseline, etc. and already have a good rapport with the patient	9	No	No		Not an outcome/standard	
Experience from stoma patients—living with a stoma	6	No	No		Not an outcome/standard	
Ease of access to prehabilitation for the patient	9	Yes	Yes	94	Already included—no further action required	
Affordability of prehabilitation for the patient	9	No	No		Not an outcome/standard	
The patient is key to the content/design of their prehabilitation programme	9	Yes	Yes	14	Already included—no further action required	

## Appendix

**Appendix 4 Description of core standards consensus meeting discussions**

When discussing the setting for prehabilitation, HCPs felt that the localization of the settings was too specific and that patients need different options depending on distance to the hospital and access to transport. Similar themes emerged in the discussion of the medium (for example, face-to face or virtual) and in relation to the physical activity, exercise and nutrition domains. It was thought that physical function could be improved, especially in the less fit or frail, within a short space of time (consensus 2–4 weeks). Psychological support was also thought to be potentially effective after 2 weeks, but some patients may need longer (consensus 2–6 weeks). After more evidence for the optimal duration of nutrition optimization was introduced, the group wanted to reflect the wide range of surgical colorectal pathologies included and agreed on a nutrition duration of 4–6 weeks.

The group moved on to talk about how patients value variation and choice in prehabilitation trials. This is demonstrated by the PPI comments: ‘not one-size-fits-all’, ‘[prehabilitation should be] tailored to your needs’, the group agreed that prehabilitation research cannot focus on just one programme and there should be consideration towards the needs of the individual. The clinical term ‘exercise prescription’ was introduced and all participants, both patients and HCPs, appreciated this individualized approach. The group also repeated that virtual or distant prehabilitation programmes were an option that could engage wider recruitment from harder to reach populations.

Patients reported that they found the term high-intensity interval training (HIIT) intimidating. HIIT was then described in detail with supporting evidence, and although HIIT was not specifically included, there was consensus that aerobic training and anaerobic training should be included. This allows future research to include any type of exercise that can provide overload, but the chair did stress that key standards are not prescriptive and are typically ‘what should be included’ not the ‘how it should be included’. Finally, the importance of prehabilitation not being a sole entity was strongly supported and that it should flow into established programmes like ERAS and rehabilitation.

## Appendix

**Appendix 5 Description of ‘outcomes’ consensus meeting discussions**

The chair stated that some outcomes were already included in other published core outcomes sets (COS) relevant to colorectal surgery. For example, the COS for colorectal cancer surgery (which included cancer recurrence), inflammatory bowel disease and for recovery of the bowel after surgery^26–28^. The group agreed that future trials of prehabilitation for colorectal surgery should also consider including the relevant condition-specific COS where applicable.

Length of hospital stay was a borderline item. Although the group acknowledged this outcome is commonly included in research studies, they voted to exclude it because it has many influencing factors and is not a direct marker of prehabilitation success. In contrast, length of critical care was thought not to be as susceptible to these influences and achieved consensus.

Discussion of physical function items produced contrasting views on the role of invasive and non-invasive tests (cardiopulmonary exercise test *versus* 6-minute walk test). To aid discussion, the chair steered the group to achieve consensus on what should be measured, rather than specifying what measurement to use. Alternate wording was proposed through group discussion with the following reaching consensus: ‘any suitable objective measure of physical function’ and ‘any suitable objective measure of cardio-respiratory function’ (physical function domain).

The outcome ‘return to normal activities’ was modified after discussion to ‘return to normal physical activity’ and was moved to the physical function domain. Postoperative course after hospital discharge was voted out, although it had achieved consensus-in previously. The group considered it to be covered by other more specific items, such as discharge destination and support requirements. For simplicity, discharge destination and support were then voted to be combined into one item with family/carer support (resource use domain).

The items ‘planned treatment does not go ahead’, ‘inability to complete physical tests’ and ‘prehabilitation stopped’ were considered and achieved consensus-out as they represented process measures rather than outcomes.

Patient activation measures (PAMs) were explained to all participants because many were unfamiliar. However, discussion reflected broad agreement that PAMs were important and valuable with voting achieving consensus.
