# Corrigendum: Defining standards and core outcomes for clinical trials in prehabilitation for colorectal surgery (DiSCO): modified Delphi methodology to achieve patient and healthcare professional consensus

**DOI:** 10.1093/bjs/znae201

**Published:** 2024-08-02

This is a corrigendum to: Rebecca Fish, Sue Blackwell, Stephen R Knight, Sarah Daniels, Malcolm A West, Iona Pearson, Susan J Moug, DiSCO Study Group , Defining standards and core outcomes for clinical trials in prehabilitation for colorectal surgery (DiSCO): modified Delphi methodology to achieve patient and healthcare professional consensus, *British Journal of Surgery*, Volume 111, Issue 6, June 2024, https://doi.org/10.1093/bjs/znae056.

The following changes have been made to the originally published manuscript. Two names have been added to the DiSCO Study Group under section **Collaborators**: Glen Guerra, and Efstratia Baili.

