# Supplementary material for: Defining standards and core outcomes for clinical trials in prehabilitation for colorectal surgery (DiSCO): modified Delphi methodology to achieve patient and healthcare professional consensus
Source: Br J Surg. 2024 Jun 18;111(6):znae056. doi: 10.1093/bjs/znae056 (PMC11185089; doi:10.1093/bjs/znae056)
Supplement: znae056_Supplementary_Data [file znae056_supplementary_data.docx]

**Supplementary Table 1:** Summary of DiSCO Delphi results through Rounds 1 and 2 and the Consensus days.

| **DOMAIN** | **Subdomain** | **ITEM** | **ID** | **Delphi R1** | **Delphi R2** | **Consensus meeting** | **Final key standards and core outcomes** |
| --- | --- | --- | --- | --- | --- | --- | --- |
| Components of Prehab |  | Exercise/ physical activity | 1 | Yes | n/a | n/a | Exercise |
|  |  | Nutrition | 2 | Yes | n/a | n/a | Nutrition |
|  |  | Psychological support | 3 | Yes | n/a | n/a | Psychological (emotional) support |
|  |  | Comprehensive geriatric assessment (for older; frail patients) | 4 | Yes | n/a | n/a | Comprehensive geriatric assessment (for older; frail patients) |
|  |  |  |  |  |  | | |
| Setting for Prehab |  | In secondary care (the hospital) **#** | 5 | Consensus-out | Consensus-out | Yes | Multi-centre |
|  |  | In primary care (the GP's practice) **#** | 6 | Consensus-out | Consensus-out | Yes |  |
|  |  | In the community; for example at a local gym or community centre**#** | 7 | Consensus-out | Consensus-out | Yes |  |
|  | | | | | | | |
| Exercise/ Physical Activity | Medium | Face-to face exercise supervision and advice**#** | 8 | Consensus-out | Consensus-out | Yes | Choice of face to face or remote |
|  |  | Remote exercise supervision and advice (e.g. by telephone or video-call) **#** | 9 | Consensus-out | Consensus-out | Yes |  |
|  | Group size | One-to-one exercise supervision and advice | 10 | Consensus-out | Borderline | Yes | Choice of one-to-one or group |
|  |  | Group exercise supervision and advice**#** | 11 | Consensus-out | Consensus-out | Yes |  |
|  | Personalisation | A personalised exercise programme specifically tailored to the individual | 12 | Consensus-out | Borderline | Yes | A personalised exercise programme specifically tailored to the individual |
|  |  | A standardised exercise programme designed for prehab but not specifically tailored to each individual**#** | 13 | Consensus-out | Consensus-out | No |  |
|  |  | General exercise advice not specifically designed for prehab | 14 | Consensus-out | Consensus-out | n/a |  |
|  | Type | Exercise that becomes progressively harder | 15 | Consensus-out | Consensus-out | n/a |  |
|  |  | High intensity/interval training | 16 | Consensus-out | Consensus-out | n/a |  |
|  |  | Endurance | 17 | Consensus-out | Consensus-out | n/a |  |
|  |  | Pulmonary physiotherapy exercises | 18 | Consensus-out | Borderline | No |  |
|  |  | Functional activity training | 19 | Consensus-out | Borderline | Yes | Functional activity training |
|  |  | Cardiovascular /aerobic exercise | 20 | Consensus-out | Borderline | Yes | Cardiovascular /aerobic exercise |
|  |  | Resistance/weight training | 21 | Consensus-out | Consensus-out | n/a |  |
|  |  | Stretching/flexibility exercise | 22 | Consensus-out | Borderline | No |  |
|  | Duration | The exercise programme should last up to 2 weeks**#** | 23 | Consensus-out | Consensus-out | No |  |
|  |  | The exercise programme should last 2-4 weeks | 24 | Consensus-out | Borderline | Yes | The exercise programme should last 2-4 weeks |
|  |  | The exercise programme should last 4-6 weeks | 25 | Consensus-out | Borderline | No |  |
|  |  | The exercise programme should be in excess of 6 weeks**#** | 26 | Consensus-out | Consensus-out | No |  |
|  |  |  |  | | | | |
| Nutrition | Medium | Face-to face nutritional advice | 27 | Consensus-out | borderline | Yes | Choice of face to face or remote |
|  |  | Remote nutritional advice (e.g. by telephone or video-call) | 28 | Consensus-out | Borderline | Yes |  |
|  | Group size | One-to-one nutritional advice | 29 | Consensus-out | Borderline | Yes | One-to-one nutritional advice |
|  |  | Group nutritional advice**#** | 30 | Consensus-out | Consensus-out | No |  |
|  | Personalisation | A personalised nutritional advice programme specifically tailored to the individual | 31 | Consensus-out | Borderline | Yes | A personalised nutritional advice programme specifically tailored to the individual |
|  |  | A standardised nutritional advice programme designed for prehab but not specifically tailored to the individual**#** | 32 | Consensus-out | Consensus-out | No |  |
|  |  | General nutritional advice**#** | 33 | Consensus-out | Consensus-out | No |  |
|  | Duration | The nutrition programme should last up to 2 weeks**#** | 34 | Consensus-out | Consensus-out | No |  |
|  |  | The nutrition programme should last 2-4 weeks | 35 | Consensus-out | Borderline | No |  |
|  |  | The nutrition programme should last 4-6 weeks | 36 | Consensus-out | Borderline | Yes | The nutrition programme should last 4-6 weeks |
|  |  | The nutrition programme should be in excess of 6 weeks**#** | 37 | Consensus-out | Consensus-out | No |  |
|  |  |  |  | | | | |
| Psychological Support | Medium | Face-to face psychological support | 38 | Consensus-out | Borderline | Yes | Choice of face to face or remote |
|  |  | Remote psychological support (e.g. by telephone or video-call) | 39 | Consensus-out | Borderline | Yes |  |
|  | Group size | One-to-one psychological support | 40 | Consensus-out | Borderline | Yes | One-to-one psychological support |
|  |  | Group psychological support | 41 | Consensus-out | Consensus-out | n/a |  |
|  | Personalisation | A personalised psychological support programme specifically tailored to the individual | 42 | Consensus-out | Borderline | Yes | A personalised psychological support programme specifically tailored to the individual |
|  |  | A standardised psychological support programme designed for prehab but not specifically tailored to the individual | 43 | Consensus-out | Consensus-out | n/a |  |
|  |  | General advice on psychological support | 44 | Consensus-out | Consensus-out | n/a |  |
|  | Type | Focus on anxiety reduction | 45 | Consensus-out | Yes | n/a | Focus on anxiety reduction |
|  |  | Focus on body image including stoma concerns | 46 | Consensus-out | Yes | n/a | Focus on body image including stoma concerns |
|  |  | Relaxation techniques e.g. breathing exercises; yoga | 47 | Consensus-out | Borderline | Yes | Relaxation techniques e.g. breathing exercises; yoga |
|  |  | Mental preparedness and motivation | 48 | Borderline | Yes | n/a | Mental preparedness and motivation |
|  | Duration | The psychological support should last up to 2 weeks | 49 | Consensus-out | Consensus-out | n/a |  |
|  |  | The psychological support should last 2-4 weeks**#** | 50 | Consensus-out | Consensus-out | Yes | Psychological support should last 2-6 weeks |
|  |  | The psychological support should last 4-6 weeks | 51 | Consensus-out | Borderline | Yes |  |
|  |  | The psychological support should be in excess of 6 weeks | 52 | Consensus-out | Borderline | No |  |
|  | | | | | | | |
| Comprehensive Geriatric Assessment |  | Cognitive assessments | 53 | Yes | n/a | n/a | All components of the comprehensive geriatric assessments |
|  |  | Medication optimisation | 54 | Yes | n/a | n/a |  |
|  |  | Co-morbidity review | 55 | Yes | n/a | n/a |  |
|  |  | Falls advice | 56 | Yes | n/a | n/a |  |
|  |  | Advanced care planning | 57 | Yes | n/a | n/a |  |
|  | | | | | | | |
| Recipients of Prehab | Reason for surgery | Patients undergoing surgery for benign conditions | 58 | Consensus-out | Yes | n/a | All types of colorectal surgery for any condition, including Patients having neoadjuvant chemotherapy |
|  |  | Patients undergoing surgery for cancer | 59 | Yes | n/a | n/a |  |
|  | Surgical approach | Patients undergoing laparoscopic (keyhole) surgery | 60 | Consensus-out | Yes | n/a |  |
|  |  | Patients undergoing open surgery | 61 | Yes | n/a | n/a |  |
|  | Neoadjuvant treatment | Patients undergoing chemotherapy or radiotherapy prior to surgery | 62 | Yes | n/a | n/a |  |
|  | Stoma | Patients having a stoma formed as part of surgery | 63 | Yes | n/a | n/a |  |
|  | Age of patient | Patients under 60 years of age | 64 | Borderline | Yes | n/a | Patients of any age |
|  |  | Patients aged 60-69 | 65 | Yes | n/a | n/a |  |
|  |  | Patients aged 70-79 | 66 | Yes | n/a | n/a |  |
|  |  | Patients aged 80-89 | 67 | Yes | n/a | n/a |  |
|  |  | Patients aged 90 and over | 68 | Yes | n/a | n/a |  |
|  | Comorbidities and risk factors | Frail patients | 69 | Yes | n/a | n/a | Patients with any co-morbidities and additional risk factors |
|  |  | High-risk' patients | 70 | Yes | n/a | n/a |  |
|  |  | Malnourished/underweight patients | 71 | Yes | n/a | n/a |  |
|  |  | Obese patients | 72 | Yes | n/a | n/a |  |
|  |  | Patients with recent or long-term mental illness | 73 | Yes | n/a | n/a |  |
|  | | | | | | | |
| Delivery of Prehab |  | Surgeon | 74 | Consensus-out | Consensus-out | n/a |  |
|  |  | Anaesthetist | 75 | Consensus-out | Consensus-out | n/a |  |
|  |  | Specialist nurse | 76 | Yes | n/a | n/a | Specialist nurse |
|  |  | Oncologist (medical or clinical) | 77 | Consensus-out | Consensus-out | n/a |  |
|  |  | Exercise physiologist or sports scientist | 78 | Consensus-out | Yes | n/a | Exercise physiologist or sports scientist |
|  |  | Exercise oncologist | 79 | Consensus-out | Borderline | No |  |
|  |  | Sports medicine specialist | 80 | Consensus-out | Consensus-out | n/a |  |
|  |  | Exercise/activity specialist e.g. a personal trainer | 81 | Consensus-out | Borderline | No |  |
|  |  | Physiotherapist | 82 | Borderline | Yes | n/a | Physiotherapist |
|  |  | Nutritionist/dietician | 83 | Yes | n/a | n/a | Nutritionist/dietician |
|  |  | Geriatrician | 84 | Consensus-out | Borderline | No |  |
|  |  | Pharmacist | 85 | Consensus-out | Consensus-out | n/a |  |
|  |  | Psychologist | 86 | Borderline | Yes | n/a | Psychologist |
|  |  | General practitioner (GP) | 87 | Consensus-out | Consensus-out | n/a |  |
|  |  | Other patients who are having/have had colorectal surgery | 88 | Consensus-out | Borderline | Yes | Other patients who are having/have had colorectal surgery |
|  | | | | | | | |
| Outcomes of Prehab | Physical musculoskeletal function | Daily or weekly Step count# | 89 | Consensus-out | Consensus-out | Yes | A suitable objective measure of physical function |
|  |  | Sit-to-stand | 91 | Consensus-out | Borderline |  |  |
|  |  | 6 minute walk test | 92 | Consensus-out | Borderline |  |  |
|  |  | Handgrip strength | 95 | Consensus-out | Borderline |  |  |
|  |  | Leg strength (e.g. leg/ quadriceps extension) | 96 | Consensus-out | Borderline |  |  |
|  | Cardio-respiratory function | Respiratory/breathing measurements e.g. peak flow | 93 | Consensus-out | Borderline | Yes | A suitable physiological measure of cardiorespiratory fitness |
|  |  | CPET | 90 | Consensus-out | Borderline |  |  |
|  |  | Pulse wave velocity* | 119 |  | Borderline |  |  |
|  | Metabolism and nutrition | Percentage body fat | 97 | Consensus-out | Consensus-out | n/a |  |
|  |  | Weight change | 98 | Consensus-out | Consensus-out | n/a |  |
|  |  | Energy expenditure | 99 | Consensus-out | Consensus-out | n/a |  |
|  |  | Change in nutritional assessment | 100 | Consensus-out | Yes | n/a | Change in nutritional assessment |
|  | Psychiatric/  Emotional functioning/  wellbeing | Cognitive issues | 111 | Borderline | Yes | n/a | Cognitive issues |
|  |  | Anxiety | 102 | Borderline | Yes | n/a | Anxiety |
|  |  | Depression | 103 | Consensus-out | Yes | n/a | Depression |
|  |  | Stoma concerns | 104 | Consensus-out | Yes | n/a | Stoma concerns |
|  |  | Stress | 105 | Consensus-out | Yes | n/a | Stress |
|  | General | Pain | 107 | Borderline | Yes | Yes | Pain |
|  | Physical function | Sleep | 106 | Consensus-out | Yes | Yes | Sleep |
|  |  | Bowel function | 108 | Consensus-out | Borderline | Yes | Bowel function |
|  |  | Return to normal activities | 110 | Yes | n/a | Yes | Return to normal physical activities |
|  |  | Fatigue | 101 | Borderline | Yes | Yes | Fatigue |
|  | Neoplastic | Cancer recurrence* | 122 |  | Borderline | Yes | Relevant condition-specific outcomes with reference to the relevant core outcome set where available |
|  | Survival | Survival* | 120 |  | Yes | n/a | Survival |
|  | Global quality of life and wellbeing | Overall quality of life | 109 | Yes | n/a | n/a | Overall quality of life |
|  |  | An overall measure of health and functioning e.g. WHODAS or DASI score* | 121 |  | Yes | n/a | An overall measure of health and functioning |
|  | Resource use | Length of hospital stay | 112 | Consensus-out | Borderline | No |  |
|  |  | Length of critical care stay (High dependency unit or intensive care) # | 114 | Borderline | Yes | No |  |
|  | Adverse events | Surgical complications | 113 | Yes | n/a | n/a | Relevant condition-specific outcomes with reference to the relevant core outcome set where available |
|  | Societal/carer burden | Discharge destination and support requirements# | 115 | Yes | n/a | Yes | Discharge destination and support requirements |
|  |  | Post-operative course after discharge from hospital*# | 125 |  | Yes | No |  |
|  |  | Family/carer involvement* | 126 |  | Borderline | Yes | Family/carer involvement |
|  | Process measures | Planned surgery does not go ahead# | 117 | Borderline | Yes | No |  |
|  |  | Prehabilitation stopped# | 118 | Consensus-out | Yes |  |  |
|  |  | Adherence to rehabilitation e.g. number of exercise sessions completed | 94 | Consensus-out | Borderline |  |  |
|  |  | Inability to complete physical tests# | 116 | Consensus-out | Yes |  |  |
|  | Behavioural | Changes in lifestyle behaviours* | 123 |  | Borderline | No |  |
|  |  | Patient Activation Measures* | 124 |  | Borderline | Yes | Patient Activation Measures |
